# Non-vectorial integration of intersectional short-pulse stimulation enables enhanced deep brain modulation and effective seizure control

**DOI:** 10.1038/s43856-026-01595-6

**Published:** 2026-04-21

**Authors:** Tamás Földi, Miklos Szoboszlay, Zoltán Chadaide, Bence Radics, Bálint Horváth, Endre Vecsernyés, István Langó, Péter Ráfi, Andrea Pejin, Lívia Barcsai, Gábor Kozák, Nóra Forgó, Kristóf Furuglyás, Olivér Nagy, Anett J. Nagy, Tamás Laszlovszky, Zoltán Somogyvári, Magor L. Lőrincz, Orrin Devinsky, Antal Berényi

**Affiliations:** 1 MTA-SZTE ‘Momentum’ Oscillatory Neuronal Networks Research Group, Department of Physiology, University of Szeged, Szeged, Hungary; 2 Neunos ZRt, Szeged, Hungary; 3 HCEMM-SZTE Magnetotherapeutics Research Group, University of Szeged, Szeged, Hungary; 4 Department of Pathology, University of Szeged, Szeged, Hungary; 5 Department of Neurology & Stroke, University of Tübingen, Tübingen, Baden-Württemberg, Germany; Hertie-Institute for Clinical Brain Research, Tübingen, Baden-Württemberg Germany; 6 Department of Physiology, Anatomy and Neuroscience, Faculty of Sciences University of Szeged, Szeged, Hungary; 7 Department of Computational Sciences, HUN-REN Wigner Research Centre for Physics, Budapest, Hungary; 8 Neuroscience Division, Cardiff University, Museum Avenue Cardiff, UK; 9 Department of Neurology, NYU Langone Comprehensive Epilepsy Center, NYU Grossman School of Medicine, New York, NY USA; 10 Neuroscience Institute, New York University, New York, NY USA

**Keywords:** Membrane potential, Excitability, Network models

## Abstract

**Background:**

Transcranial electrical stimulation (TES) has limited spatial focus and depth penetration, constraining its therapeutic efficacy. Intersectional Short-Pulse (ISP) stimulation was developed to overcome these limitations by delivering rapidly switching pulses that can be temporally integrated by neuronal membranes. Here, we aimed to establish the biophysical basis of ISP-induced temporal summation and to test whether this mechanism enables effective brain modulation in vivo.

**Methods:**

We combined finite-element modeling, cadaver measurements (*n* = 2 human cadavers), and biophysically realistic NEURON simulations to characterize the spatial and temporal properties of ISP-induced electric fields. In vivo whole-cell patch-clamp recordings were performed in the rat somatosensory cortex (female Wistar rat) to test the membrane-level integration of sequential electric field pulses. Functional efficacy was evaluated using closed-loop ISP stimulation in a hippocampal kindling model of temporal lobe epilepsy in male Long–Evans rats (*n* = 11 animals, >500 induced seizures analyzed across conditions).

**Results:**

Here we show that neurons integrate sequential ISP pulses in a non-vectorial, temporally accumulative manner, consistent with membrane-level charge integration rather than extracellular field superposition. ISP and conventional TES simulations produced similar instantaneous field magnitudes, but ISP stimulation resulted in more uniform neuronal excitability across brain depths. Closed-loop ISP stimulation significantly outperformed conventional TES in reducing seizure duration and severity. ISP reduced hippocampal seizure duration by 45% and 35% compared to SHAM stimulation and conventional TES, and significantly reduced motor seizure severity.

**Conclusions:**

ISP stimulation provides a non-invasive neuromodulation approach that enhances deep brain engagement through rapid, temporally structured pulse sequences. These findings demonstrate effective seizure suppression in a rodent model and support the translational potential of ISP for disorders involving pathological neural dynamics.

## Abstract (web summary)

Földi, Szoboszlay et al. evaluate Intersectional Short-Pulse (ISP) stimulation, a transcranial brain-stimulation approach, through modelling, cadaver measurements, intracellular recordings, and in vivo testing in a rat model. ISP stimulation shows strong epileptic seizure-suppressing effects and improves deep-brain engagement.

## Plain language summary

Epilepsy is a disorder in which sudden bursts of abnormal brain activity cause seizures. Treatments do not work for everyone, and many patients need safer ways to calm this activity without surgery. In this study, we tested a new form of electrical stimulation called Intersectional Short-Pulse (ISP) stimulation. It delivers very brief electrical pulses through different pairs of scalp electrodes in rapid sequence.

We first used computer models, laboratory measurements, and recordings from individual brain cells of rats to understand how the brain responds to these pulses. We then tested ISP stimulation in rats with epilepsy.

ISP stimulation quickly reduced seizure activity and made severe seizures much less common. These results suggest that ISP could become a non-invasive option to control seizures and may help guide future treatments for people with difficult-to-treat epilepsy.

## Introduction

TES is a widely used non-invasive technique to modulate brain activity, a potential therapy for neurologic and psychiatric disorders^1–13^. By inducing weak EFs in the brain, TES can alter neuronal membrane potentials (V_m_) and influence spike timing and network oscillations^11,14–21^. However, the clinical efficacy of conventional TES is limited by poor spatial focality, limited penetration depth, and peripheral side effects at higher intensities^22–24^.

Another technical challenge in TES is the “mirror effect,” where simultaneous activation of surface electrodes induces opposing effects on brain activity under the cathode and anode^25^. This is particularly problematic when targeting spatially distributed oscillations generated by long-range neuronal networks, as in epileptic seizures^1,26–30^. Electrode configurations to address this issue fail to eliminate the mirror effect or overcome the trade-off between focality and depth of stimulation^31^.

Innovations in TES have increasingly focused on enhancing the spatial precision and depth targeting of applied EFs. Spatial summation of multiple current sources is a promising strategy, exemplified by methods like high-definition tDCS (HD-tDCS) and Temporal Interference (TI) stimulation. HD-tDCS employs arrays of small electrodes to confine the EF to targeted cortical areas, improving focality and minimizing off-target effects^31–35^. TI stimulation, introduced by Grossman et al., generates low-frequency EFs at specific brain regions through interference patterns of high-frequency currents, enabling constraining of the interference part of the stimulation (i.e., the beat frequency) to deeper brain regions^36–39^.

Both HD-tDCS and TI represent substantial advancements in spatially directing the effects of brain stimulation. However, achieving higher stimulation intensities for enhanced deep-brain modulation while minimizing peripheral and off-target side effects remains an ongoing challenge.

Using the time-integration principle, ISP stimulation^22^ was introduced to increase intracerebral field strength while reducing the adverse peripheral effects. Unlike conventional approaches constrained by the superposition principle of electric fields in the extracellular space, ISP is hypothesized to induce neuronal activation through the temporal integration of successive transmembrane charge injections, a form of scalar integration by the neuronal membrane, rather than through the vectorial (Maxwellian) summation of simultaneously applied fields, as for example in TI stimulation. This distinction forms the central mechanistic hypothesis of the present study, which tests whether temporally separated field pulses can elicit effective neuronal activation without relying on instantaneous vectorial summation. This principle enables non-invasive brain stimulation with greater spatial precision and improved neuromodulatory efficacy in both research and clinical contexts.

To test the predictions of the ISP hypothesis, we combined computational modeling, cadaver measurements, in vivo electrophysiology, and a rodent model of temporal lobe epilepsy. We provide evidence for the mechanism of ISP stimulation via membrane integration in in vivo intracellular recordings and demonstrate its superior efficacy in controlling epileptic brain activity in a rat model.

## Methods

### Conceptual framework of ISP stimulation

**Fig. 1:**
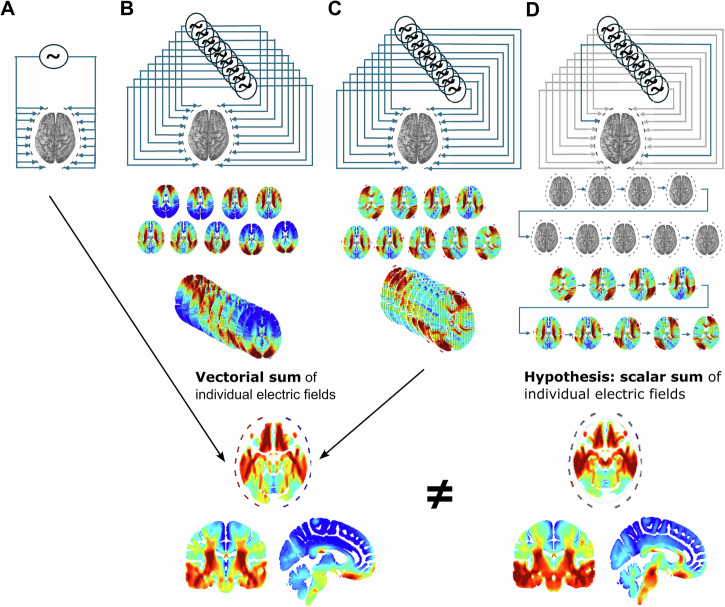
Equivalence of EF distributions in TES under different source configurations. This figure illustrates the principle that the EF distribution in the brain is determined by net current flow, regardless of the source configuration. Three scenarios are presented: **A** A single common source driving all electrodes simultaneously, similar to HD-tDCS. **B** Multiple independent sources applied simultaneously with non-crossing current paths, similar to TI stimulation. **C** Multiple independent sources applied simultaneously with crossing current paths. The top row shows schematic representations of the electrode placements and current paths of the individual sources for each scenario. The bottom row displays the simulated EF distributions in axial, coronal, and sagittal views. All three scenarios produce identical EF distributions within the brain since the principle of superposition dictates a geometric (i.e., vectorial) net effect as the sum of independent sources if the total current input at each electrode is equivalent across scenarios. In contrast, if the independent sources are sequentially but not concurrently activated (**D**), the EFs remain discreet, and integration occurs intracellularly as charge accumulation. Due to the non-linearity of the transmembrane charge injection, we hypothesize that this arrangement results in a different neuronal ‘readout’, which more closely resembles the algebraic (i.e., scalar) sum of individual EFs.

Maxwell’s theory of electromagnetism and the principle of superposition hold that the EF distribution within a conductive volume (e.g., the head) is determined by the net current flow, whether this current originates from multiple independent sources or a single common source connected to multiple electrodes. For TES, when multiple independent current sources are attached to the scalp, the EF distribution and strength within the head are identical to what would be produced by a single source connected to the same electrode montage if the net current input at each electrode location is the same in both scenarios. Thus, using multiple independent current sources instead of a single source with multiple electrodes does not improve the EF’s focus or shape in the brain (Fig. 1; Supplementary Fig. 1). Any technique that enhances spatial focality must rely on principles or methods beyond merely increasing the number of independent current sources.

An alternative to the spatial summation is to exploit the temporal integrative properties of the neuronal membrane. When multiple source effects are time-integrated by neurons by applying independent sources in rapid succession rather than simultaneously (Fig. 1D), each discrete extracellular EF injects current across the neuronal membrane. Since transfer across the cell membrane is non-linear, neurons can integrate individual charge injections induced by these rapidly successive, quasi-independent source effects.

The principle of intracellular integration has several implications. The extracellular space is largely an ohmic conductor, causing the extracellular fields to build up and disappear instantaneously. By applying EFs in rapid succession rather than simultaneously, each field induces its discrete charge injection before the next field is applied. The cell membrane’s capacitive properties enable it to accumulate charge over time, even from fields that would normally cancel in the extracellular space due to their vectorial summation by superposition. Furthermore, the non-linear characteristics of the membrane’s transfer function may amplify or modulate the integration of charge injections in ways that are not predictable from linear superposition.

### Model neurons and E-field stimulation of model neurons

We adjusted models of neurons with realistic axonal and dendritic morphologies for simulations of the single-cell response to stimulation using the EF #15 (basket) and #20 (pyramidal) cells^40^. The field intensity (ISP intensity) was scaled to determine the threshold to evoke one AP with a whole sequence of ISP pulses of six different pairs of sources. We used NEURON software^41^ and customized Python (3.8.8 on macOS Sonoma) scripts to model the effect of local extracellular fields with gradually changing intensities and directions (representing the local effects of the consecutive ISP pulses).

### Simulating transcranial electric stimulation

The finite element model for EF simulation was created from rat head MR scans using an open-source implementation of the Realistic Volumetric-Approach to Simulate Transcranial Electric Stimulation (ROAST) modeling software^42,43^. Models are constructed based on magnetic resonance images (MRI) of the rat head^44^ and the human head for a detailed representation of the three-dimensional anatomy. To simulate current flow on an MRI, virtual electrodes are located on this anatomical model, the volume is tessellated into a mesh, and the FEM is solved numerically to estimate the current flow. ROAST combines the segmentation algorithm of SPM12, a Matlab script for touch-up and automatic electrode placement, the finite element mesher iso2mesh, and the solver getDP^42^. In our FEM, the simulated electric potential spans positive and negative values centered around zero, because ROAST (through getDP) uses an arbitrary internal reference—often within the brain volume itself. By contrast, our in vivo measurements are referenced to a distant electrode, introducing a substantial common‑mode (DC) offset.

### Cadaver recordings

The cadaver experiments were approved by the Regional and Institutional Review Board of Human Investigations in the University of Szeged (22/2023-SZTE) and the Medical Research Council - Scientific and Research Ethics Committee of Hungary (BM/18167-3/2023). Two cadavers were investigated in this study. Cadavers were selected based on the absence of significant skull or brain abnormalities and recent anatomical imaging (CT or MRI) to ensure accurate modeling of electric current flow. After positioning the cadaver prone on the autopsy table, standard skull measurements were recorded, and a 10-20 EEG electrode cap was used to mark the electrode entry points. Four electrode strips (Ad-tech TS08R-SP10X-000) were implanted subgaleally through small incisions, secured with sutures, and verified by palpation. A stereotactic frame was attached symmetrically to the skull using ear bars, and the stereo EEG (sEEG; Medtronic 3389-28) lead insertion was guided by predefined trajectories planned via merged cadaver imaging with the stereotactic frame model. The sEEG leads were stained with ink to mark the electrode tracks for post-experimental track reconstruction, ensuring accurate analysis of their trajectories in subsequent histological evaluations. Positions of the sEEG leads and the subgaleal strips were verified by a C-arm X-ray. Electrical recordings were obtained at multiple depths.

ISP stimulation was delivered through the subgaleal strip electrodes using the Neunos Neuroclinical device (Neunos, Szeged, Hungary), while biosignal recordings were performed from the sEEG electrodes with the Amplipex KJE-1001 data acquisition system (Amplipex, Szeged, Hungary), with a sampling rate of 1.28 MS/s per channel and no signal filtering. Impedance checks were performed at each depth, and predefined ISP stimuli were delivered at the strip electrodes.

After the recordings, the brain was removed and fixed in a paraformaldehyde bath for at least two weeks before sectioning. Macroscopic analysis of the sEEG lead trajectories was conducted on coronal slices of the fixed brain. The electrode and lead positions were reconstructed using pre-mortem imaging data and Slicer3D software (https://www.slicer.org/).

### Animals

All experiments were performed following European Union guidelines (2003/65/CE) and the National Institutes of Health Guidelines for the Care and Use of Animals for Experimental Procedures. The Ethical Committee approved the experimental protocols for Animal Research at the Albert Szent-Györgyi Medical and Pharmaceutical Center of the University of Szeged (XIV/824/2021). Eleven male Long-Evans rats were used in the experiments. Animals were group-housed (2–3 per cage) prior to surgery and individually housed after surgery for implant protection and monitoring. Housing conditions and environmental enrichment followed standard institutional and ethical guidelines.

### Surgery

The animals were operated on under isoflurane anesthesia, followed by standard postoperative care and systemic analgesia using non-steroidal anti-inflammatory drugs (NSAIDs), administered in accordance with institutional and ethical guidelines^45^. The rats were implanted with recording electrode triplets (inter-wire spacing, 0.4 mm)^46^ the frontal areas (AP: +2 mm from the bregma; ML: +2 mm in the sagittal plane; DV: −1,4 mm from the dura) and the dorsal hippocampus (AP: −4 mm from the bregma; ML: + 0.7 mm, +2.7 mm, +4 mm in the sagittal plane; DV: −3 mm from the dura) bilaterally. Five pairs of #000 diameter (0.86 mm) stimulation stainless steel screw electrodes were implanted over the temporal bone bilaterally. Miniature stainless-steel screws (serving as reference and ground) were implanted above the cerebellum^46^. A bipolar stimulus electrode consisting of two insulated tungsten wires (inter-wire spacing, 0.5 mm) was prepared for kindling and seizure induction. Insulations were stripped 0.4-0.5 mm around the tips to decrease their impedance to 10-20 kΩ at 1 kHz. This stimulus electrode was implanted targeting the hippocampal commissure (AP: −0.84 mm from the bregma; ML: 1.5 mm, angled at 18° from the sagittal plane; DV: −1.5 mm). A copper mesh (acting as a Faraday cage) was built around the electrode holders and secured with dental cement^46^. The rats were individually housed after the implantation, and were monitored regularly for signs of pain, distress, or postoperative complications. No unexpected adverse events occurred during the study. Details of the perioperative care and analgesia, as well as the applied humane termination endpoints, are detailed in our previous publication^45^.

### Hippocampal electrical kindling

After recovery from the implantation surgery, the HPC was stimulated every day at subconvulsive intensity via the kindling electrode. Each kindling stimulation included 120× 0.5 ms positive – 0.5 ms negative bipolar rectangular pulses at 62.5 Hz^47^ delivered by an isolated stimulator generator in voltage control mode (STG4008; Multi-Channel Systems). Stimulus intensity was set as the minimum induced after-discharge (10-25 Hz population spikes synchronously recorded in > 50% of HPC channels after the HPC commissure stimulation), commonly ±600-3000 mV. The kindling stimulation was performed six times per day for ten days. The interstimulus intervals were at least 30 minutes.

### Data acquisition from freely moving rats

Local field potential (LFP) recordings were made in the animals’ home cages, and food and water were given *ad libitum*. The recording wire electrodes were connected to a signal multiplexing headstage (HS3_v1.3, Ampliplex) and stored after digitalization at a 500 Hz sampling rate (KJE-1001, Ampliplex)^48^. In parallel, rats’ preamplified signals were analyzed online by a programmable digital signal processor (RX-8, Tucker-Davis Technologies, FL, USA) using a custom-made seizure detection algorithm^45^.

### Closed-loop transcranial stimulation

HPC electrographic seizure waves were detected online using a programmable digital signal processor. These detected waves triggered the transcranial stimulation for on-demand real-time seizure interventions^45^. The LFP signals were analyzed online to detect LFP deflections in the HPC using a custom-made seizure detection algorithm based on a previously developed method. Briefly, the pre-selected HPC channel was band-pass filtered with a 4^th^-order Butterworth filter, rectified, and integrated into a time window. Each transcranial stimulus was triggered by an ictal HPC LFP deflection when the filtered signal exceeded a threshold. The time resolution of the detection was 2 ms.

Synchronous multiple threshold crossings triggered a trapezoidal single-pulse (0.5 ms; 1 ms; 5 ms; 15 ms; 80 ms) stimulation (STG4008; Multi-Channel Systems, Germany). Stimulation electrode impedances were monitored using NanoZ (Multi-Channel Systems, Germany). The stimulation was performed in voltage mode. In both conventional TES and ISP mode (see ‘Introduction’ section), the stimulus intensity was set to 8000 mV, providing approximately 200–400 µA stimulation intensity. For ISP stimulation, each electrode pair was pulsed for 100 μs, and the pulses cycled through the three pairs. This sequence was repeated to stimulate the right or left hemisphere alternately. Diffuse stimulation was applied between the left and right temporal electrodes. On each experimental day for the closed-loop intervention trials, we performed at most six recorded induced seizures. At most, two sessions of each type (SHAM, ISP, diffuse) were conducted. The order of types was randomly chosen daily. For the ISP pulse duration parameter optimization experiment, we tested every predetermined parameter daily in random order.

### Behavioral monitoring

The rats’ behavior was continuously monitored during the electrophysiological recordings with a webcam (LifeCam Studio) and synchronized with the recorded neuronal data. The severity of motor seizures was estimated online and offline based on Racine’s scale for seizures: 1, mouth and facial movements; 2, head nodding; 3, forelimb clonus; 4, rearing; 5, rearing and falling^49^.

### Histology

After data acquisition, electrolytic lesions were made at the electrodes’ tip to verify their location and possible pathologic changes. Rats were deeply anesthetized with 1.5 g/kg urethane (intraperitoneal) and transcardially perfused with saline, followed by 4% paraformaldehyde (PFA) and 0.2% picric acid (PA) in 0.1 M phosphate buffer. Brains were post-fixed overnight, and 50 μm-thick coronal sections were prepared with a vibratome (VT100S Vibratome, Leica) and stained with 4’,6-diamino-2-phenylindole dihydrochloride (DAPI; D8417, Sigma-Aldrich).

### In vivo whole-cell patch-clamp recordings with concurrent transcranial electrical stimulation

A 3-month-old female Wistar rat was anesthetized with urethane and was implanted with recording electrode triplets as described above. A craniotomy of 1 mm in diameter was drilled above the somatosensory cortex of the right hemisphere to provide access to the brain while leaving the dura intact. A small sink was built from dental cement surrounding the craniotomy and was filled with ACSF to prevent the tissue from drying and to hold the ground electrode in place. Pipettes were pulled from thick-walled borosilicate glass (BF150-86-15, Sutter Instrument, Novato, CA) with a Sutter P-1000 puller (Sutter Instrument, Novato, CA). Pipette tip resistance ranged between 5-10 MΩ when the electrodes were filled with a K-gluconate-based internal solution (containing, in mM: 130 K–gluconate, 8 KCl, 10 HEPES, 4NaCl,4Mg-ATP, and 0.3Tris-GTP,14(tris-)phosphocreatine) with pH = 7.28 and osmolality 295 mOsm) at 32 °C). Whole-cell patch-clamp recordings were performed blindly 200-250 μm under the dura, most likely targeting L2/3 pyramidal neurons. Recordings were discarded if access resistance exceeded 60 MΩ or if the holding current at −60 mV was larger than −500 pA. Signals were amplified using a MultiClamp 700B amplifier and were digitized at a sampling frequency of 50 kHz using an Axon Digidata1440 digitizer that was controlled by pClamp 10.3 software (Molecular Devices, San Jose, CA). ISP stimulation epochs were manually delivered while in whole-cell configuration in current clamp mode at an intensity of 100 μA and a duration of 100 ms (17–18 times). The same ISP stimulation sequences were repeated extracellularly as well (20 times) to compare the induced electrical artifacts between the two configurations. Recordings were subsequently processed with the pyABF v2.3.5 Python site-package https://pypi.org/project/pyabf/ and analyzed offline by custom-written Python scripts.

### Analysis of cadaver recordings and corresponding simulations

To validate the in silico ROAST modeling of rapid transient fields induced by ISP stimulation, we compared the simulated voltage gradients with real-life measurements recorded from cadavers. The ROAST model incorporated MRI-based electrode positions and the precise ISP stimulation sequence delivered via subgaleal electrode strips, identical to those used in the cadaver measurements. This allowed for the generation of 3D potential maps, which provided simulated voltage values at predefined voxel locations corresponding to the sEEG electrode contact sites.

The sEEG electrode recordings obtained from cadaver experiments were processed to extract discrete voltage potentials for each 100-µs ISP slot. Post-processing of the recorded data involved detrending, aligning, and extracting the median values across multiple repetitions of the ISP stimulation. These extracted values were used to construct potential maps, which represented the measured voltage gradients at the sEEG electrode contact points.

The next step in validation involved mapping the measured electrode positions from the cadaver recordings to the corresponding voxel locations in the ROAST model. Pairwise voltage differences were calculated for each electrode pair, generating a voltage difference matrix for both the simulated and real-life data. To assess the accuracy of the ROAST model, we performed pairwise correlations between the simulated and recorded voltage gradients. A matrix of correlation values was created for all possible sEEG electrode pairs, allowing for a detailed comparison between the model and experimental results.

A more granular analysis was also performed to investigate the correlation between the simulated and measured values as a function of the strength of the voltage gradient and as a function of the distance between the active anodes and cathodes. It was hypothesized that smaller voltage gradients would exhibit a lower SNR, potentially diminishing the accuracy of the predictions. Additionally, it was assumed that more localized stimulation would generate strong EFs in restricted brain regions, which could result in many gradients being near zero or noisy.

### Duration of electrographic seizures and normalized seizure length

Signals sampled at 500 Hz were filtered between 1 and 250 Hz for LFP signals. Peri-stimulus LFPs (30 s baseline and 180 s test epochs) were then extracted using timestamps recorded in a digital channel. The peri-stimulus LFP signals were further band-pass filtered to 10–80 Hz to prepare narrow-band LFPs. The narrow-band LFPs were then smoothed using a three-second-long moving average filter. The duration of the HPC and CTX electrographic seizures was automatically detected and defined in test epochs when all amplitudes of the smoothed LFPs in each brain region exceeded three times the root-mean-square levels of the corresponding baseline epochs. Electrographic seizures’ durations with electrical interventions were refined by the consensus estimate of two experienced researchers doing manual inspections and using Neuroscope software (RRID: SCR_002455)^50^. This refinement was done because the automated detection algorithm sometimes misestimated durations due to electrical artifacts. The variation between the manual estimates of both researchers and the automated detection data of ISP interventions was less than five percent. We normalized the seizure length to the mean duration of the SHAM-stimulated seizures within each animal.

### Assessment of the severity of motor seizures

The severity of motor seizures was video-monitored and evaluated according to Racine’s scale on each trial.

### Statistics and Reproducibility

All in vivo experiments were performed with biological replication across animals. For in vivo seizure experiments, each animal constituted one biological replicate. Within each animal, multiple seizures were recorded under each condition (SHAM, diffuse stimulation, and ISP), providing technical replication for within-animal comparisons. Sample sizes were selected based on prior literature using similar seizure and neurostimulation paradigms and established field practice. No formal a priori power calculation was performed, as the study was designed to test mechanistic principles using within-animal comparisons while minimizing animal use. Sample sizes are reported as the number of animals (biological replicates), and the total number of seizures recorded per condition is provided in Supplementary Data 1. All stimulation conditions were interleaved in a pseudorandom order within animals to minimize potential confounding effects of time or seizure history. The investigators were blinded during the evaluation of the SHAM and ISP stimulation effects in rats. No blinding or randomization was employed in the other experiments.

All data acquired were included in the analyses, and no experiments or data points were excluded post hoc based on outcome. All data analyses were performed in MATLAB (RRID: SCR_001622; Mathworks, Natick, MA, USA) and Python 3.8 unless otherwise noted. Values are expressed as mean ± standard deviation (SD) unless otherwise stated. MATLAB’s Statistics Toolbox^51^ was used for the statistical tests. Analyses were conducted under an a priori assumption of approximate normality of seizure duration measures. Data distributions were assessed by visual inspection and symmetry checks. For comparisons between stimulation conditions, a two-sample t-test with Bonferroni correction was used based on the per-seizure data, unless otherwise noted. The significance level was set at *p* < 0.05. One, two and three asterisks on the figures indicate significance levels <0.05, <0.01, and <0.001, respectively.

## Results

### Validation of ROAST to high-frequency ISP stimulation

ROAST (Realistic volumetric approach to simulate transcranial electric stimulation) is an established finite element modeling software that models the intracerebral electric potentials and potential gradients (i.e., EFs) during quasi-static tDCS-like TES^42,43^. While the primary ohmic conductance of the brain’s extracellular space is demonstrated in the sub-MHz frequency range^22^, ROAST has not been validated for ISP with instantaneous EFs with rapidly changing pulsed stimuli.

**Fig. 2:**
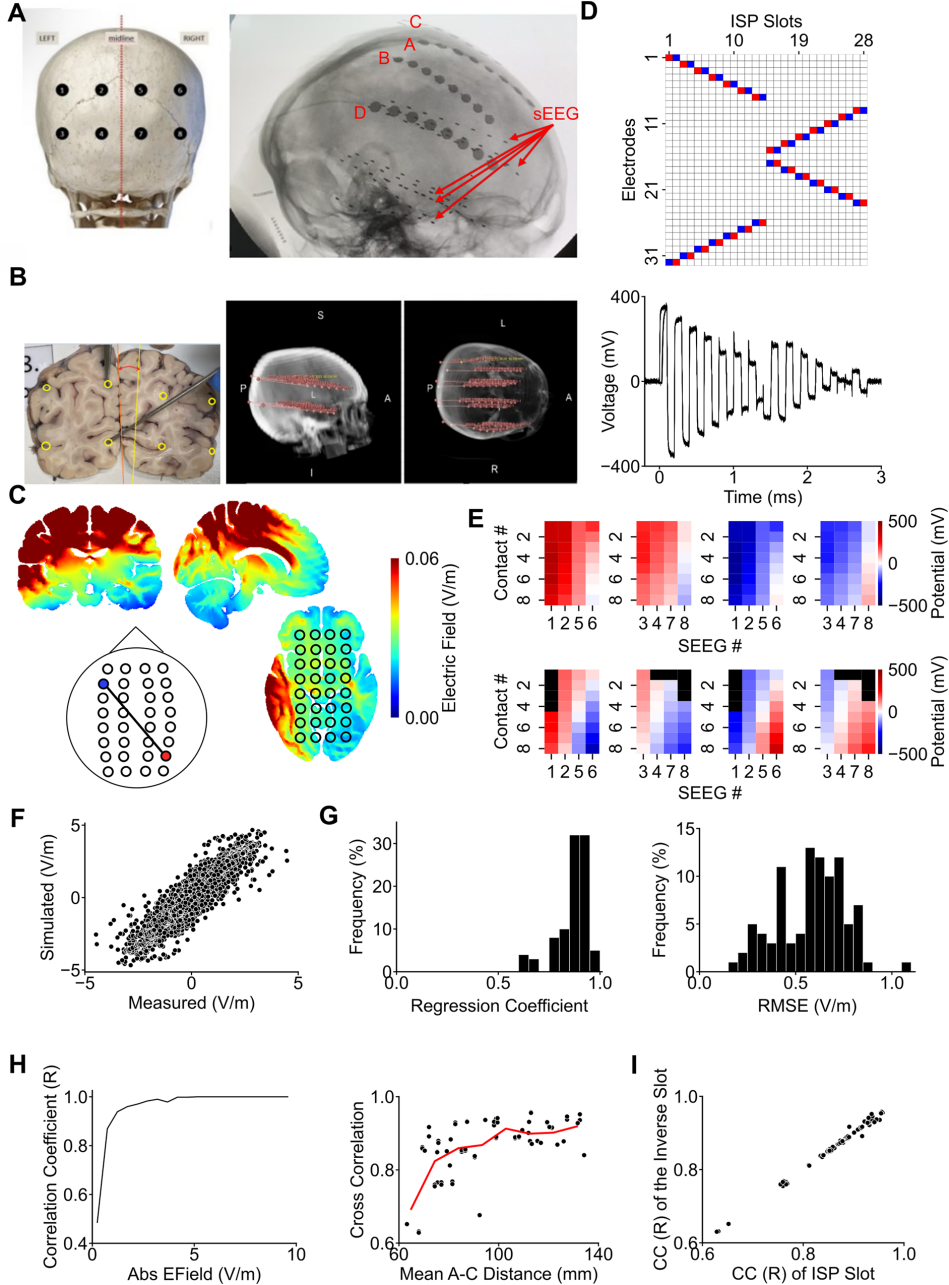
Validation of ROAST finite element modeling for ISP stimulation in human cadavers. **A** Experimental arrangement of simultaneous intracerebral sEEG recording and transcranial ISP stimulation in human cadavers. Four eight-contact stimulation strips, labeled C, A, B, and D from left to right, are implanted subgaleally, parallel to the midline. Contact points of each electrode strip were numbered from one to eight, from the front to the back. **B** An example brain slice of the post-experimental tissue processing, showing the stained electrode tracks used for post-hoc reconstruction of the sEEG recording electrode locations. 3D image shows the 3D reconstruction of the verified recording sites on the MR image of the head. **C** An example simulated effect of one ISP slot is shown as heatmaps on the cross-sections of the three main planes of the brain. Marks on the heatmaps denote these locations, from which the simulated potential levels were extracted. Inset: Schematic representation of the four subgaleal electrode strips and the electrodes utilized by this particular ISP stimulation slot. **D** The plot shows the individual voltage readouts of one intracerebral sEEG electrode contact, superimposed on each other, during the “Central” stimulation sequence. Note that the different electrode assignments within the sequence induced distinct potential readouts throughout the stimulation sequence. The electrode assignments to each ISP stimulus pattern (‘slot’) are shown above the recording. To ensure charge balancing, every other electrode assignment is identical to the preceding assignment with the polarity flipped. Blue: cathode, red: anode, white: disconnected. **E** Measured (top row) and simulated (bottom row) potential maps generated by the simultaneous readouts of all sEEG electrodes during the first two ISP pulses shown on panel D. Potential values are re-referenced to a common average. sEEGs #1, 2, 5, 6 and #3, 4, 7, 8 correspond to the dorsal and ventral measurement planes, respectively, and thus are plotted separately. Black: excluded electrodes due to unrecoverable spatial locations in the brain. Note the similarity of the voltage gradient patterns of the corresponding measured and simulated pairs. **F** Correlation of the simulated and the measured voltage gradients of all possible sEEG electrode pairs in an example ISP Stimulation pattern (*N* = 94 pairs, *p* ˂ 0.001, Pearson’s linear correlation). **G** Distribution of regression coefficients (left panel) and RMSEs (right panel) of the correlations of the measured and simulated electrical field gradients across all ISP stimulation patterns. **H** Correlation of measured and simulated EF gradients as a function of gradient magnitude (left) and average anode-cathode distance (right). **I** Similarity of the correlation coefficients of ISP slots with identical stimulation parameters but inverted electrode polarities (*N* = 46 ISP slot pairs, *p* ˂ 0.001, Pearson’s linear correlation).

ISP stimulation sequences were studied with brief, discrete tDCS-like stimuli and varying electrode arrangements applied to two human cadaver heads through four 8-channel subgaleally implanted electrode strips (32 contacts). Intracerebral potentials were measured by eight 8-channel stereoEEG (sEEG) electrodes implanted geometrically regularly on cadaver brains through small, isolated burr-holes, maximally maintaining scalp and skull integrity (Fig. 2A).

A custom transcranial neurostimulator (Neuroclinical, Neunos Ltd, Szeged, Hungary) delivered charge-balanced sequences of ISP stimuli. Individual ISP stimulus pulses were 100 μs long; typically, 24–30 stimuli were delivered in a sequence. All ISP sequences were repeated ≥100 times, then averaged to improve the signal-to-noise ratio (SNR) of intracerebral EF recordings. Four ISP sequences were designed to target frontal, parietal, or occipital lobes or midbrain.

The exact subgaleal contact site locations used for stimulation were mapped on the international 10/10 system. The post-experimental processing of the brains reconstructed the dye-covered sEEG electrode tracks and contact site locations. Locations were transformed into recording site voxel coordinates (Fig. 2B).

The cadaver head MRIs were used to model the induced intra-cadaver brain potential distributions for each ISP stimulation slot (i.e., for each electrode assignment and stimulation arrangement) by a modified version of the ROAST modeling environment, allowing for subgaleal stimulation electrode placement. This iterative modeling approach led to a set of instantaneous potential distribution maps, one for each brief stimulus pulse in the stimulation sequence. An example potential map with an electrode assignment targeting mainly the parietal regions and the left temporal region is shown in Fig. 2C. The predicted potential levels for each sEEG contact site were extracted from the finite element models (Fig. 2E lower panels).

The induced intracerebral EFs were recorded at 64 or 80 locations by a high-speed DC-coupled electrophysiologic amplifier (KJE-1001, Amplipex, Szeged, Hungary) at a 1.28 MS/s sampling rate (Fig. 2D). Baseline shifts of the intracerebral recordings due to any direct current biopotentials were removed *post hoc*. The potential traces were discretized by reading out the potential value of each contact point at the late phases of each ISP slot to avoid the effects of switching transients.

The discretized values of all sEEG contact points were used to calculate the measured potential gradient maps for each ISP stimulation pattern, and the potential values of the corresponding voxels of the simulations were extracted (Fig. 2E). Some electrode locations were unidentifiable by the *post hoc* histological analysis; these electrode potential values were excluded from the analyses (*N* = 9 of the 64 electrodes in two cadavers). The reconstructed contact site locations informed the inter-electrode distances to calculate the EFs from the contact site potential values during the processing of the simulation and experimental data and predicted EFs.

The correlation of simulated and measured inter-electrode voltage gradient pairs (*N* = 2016 to 3160 data points per ISP pattern, *N* = 94 ISP total patterns) was calculated (RMSE; Fig. 2F). The modeled and measured voltage gradients showed a high correlation (median R = 0.8791, IQR = 0.079, *p* ˂ 0.001; median RMSE = 0.6048, IQR = 0.2613; *N* = 94 for both comparisons), also validating the ROAST model for estimating the distribution of EFs induced by transient stimulus pulses (Fig. 2G). The model consistently predicted slightly higher EFs than those measured in the cadavers (Fig. 2F, mean regression quotient = 1.48 ± 0.42×, *N* = 94 ISP slots from two cadavers, each with *N* = 2016 to 3160 data value pairs).

The detailed analysis of the data points with lower correlation revealed a larger average distance between anodes and cathodes, and larger induced EF gradients improved model predictions, while stimulation between electrodes in close proximity (e.g. between neighboring electrodes on the same electrode strip) decreased model prediction (Fig. 2H), likely reflecting stronger local tissue shunting and higher sensitivity of the model on the exact tissue segmentation and MRI resolution. The modeling framework’s reliability is also supported by the consistent correlation coefficients for ISP slot pairs where electrode polarities were swapped, but the electrode assignments were identical (Fig. 2I, Correlation of correlation coefficients: R = 0.9973, *p* ˂ 0.001; Correlation of RMSEs: R = 0.8874, *p* ˂ 0.001; *N* = 46 ISP slot pairs, in both comparisons). In conclusion, these measurements confirm the validity of ROAST as a modeling environment for the rapidly switching pulses of ISP. This makes it an appropriate tool for modeling the local EF vector sequences of ISP, facilitating further modeling of neuronal readouts.

### Modeling the neuronal response to ISP stimulation

**Fig. 3:**
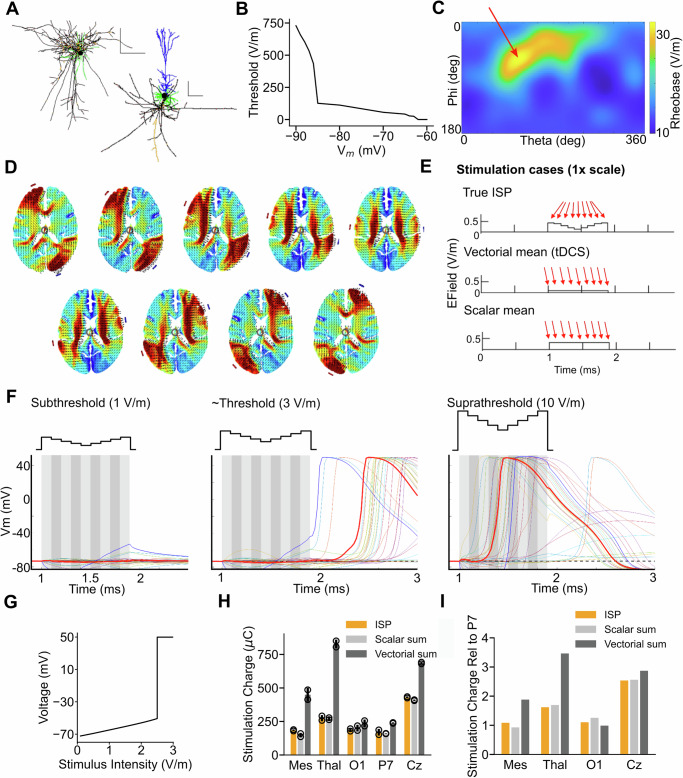
Simulation of ISP and diffuse TES effects on single neurons in the human brain. **A** Neuron model morphologies (scale bars: 50 μm). **B** The stimulus threshold to induce an AP with ISP stimulation strongly depends on the resting V_m_ of the neuron, particularly at strongly hyperpolarized states. **C** Altering the sources’ direction (i.e., anodal vs. cathodal) caused only a threefold change in the required current intensity to evoke an AP. Arrow denotes the least excitable orientation of the example neuron. **D** Modeled instantaneous EF maps of consecutive slots for a 9-slot rotating ISP sequence. **E** Magnitudes and directions of consecutive 100 µs-long EFs were used as stimuli in the three stimulation scenarios. The figure shows the gradually changing directions and magnitude of the true ISP stimulation (top) and the constant vectors applied as the vectorial (middle) and scalar mean (bottom) equivalents. **F** An example of the neuronal responses for a single 0.9 ms-long sweep of the true ISP stimulation sequence. Thick red lines denote the somatic V_m_. Note that while an EF pattern of 1 V/m average intensity failed to induce an AP, the 3 V/m fields evoked an AP at the soma 1.4 ms after stimulus offset, while a 10 V/m field evoked an instantaneous AP at the soma. Note that there is no temporal overlap between the subsequent 100 μs-long ISP pulses. **G** The highest local depolarization at any of the segments of the model neuron as a function of stimulation intensity. Note the linear relationship at subthreshold intensities. **H** The stimulus intensity threshold for ISP entrainment (orange) is similar near the electrodes (auditory cortex, AuC) and at the targeted deep crossing points (left and right hippocampi). The integrated ISP effect is best approximated with the scalar sum of individual ISP pulses (light grey) rather than their vectorial sum (dark grey), which requires substantially higher intensity to entrain deep structures than at sites proximal to the stimulating electrodes. *P* < 0.001 for ISP vs scalar sum, and *p* = 0.023 for ISP vs vectorial sum, *N* = 2 × 10 in both comparisons, Pearson’s linear correlation. **I** Same as (**G**), relative to surface-proximal structures (P7).

To investigate how individual neurons integrate ISP stimulation, we simulated neuronal responses of ‘humanized’ model neurons with realistic morphologies^40^ (Fig. 3A) by exposing them to simulated EFs generated by ISP and conventional TES stimulations. The rheobase of the neurons (i.e., minimum voltage gradient capable of inducing an action potential (AP)) was tested as a function of the resting V_m_ and neuronal orientation. We found a steep decrease in excitability when neurons were hyperpolarized below −86 mV, a close to linear relationship between −63 and −86 mV, and sporadic spontaneous APs above −62 mV (Fig. 3B). Neuronal excitability depended on the orientation of the neurons, resulting in an ~3.5-fold difference (median = 3.57, IQR = 7.93, *N* = 25) in the rheobase between the maximal and minimal excitable orientations (example neuron on Fig. 3C: 32 V/m at θ = 120° and φ = 60° vs. 10 V/m at θ = 270° and φ = 90°, 0.9 ms-long square wave stimulus, L2/3 pyramidal neuron, resting V_m_ −73 mV). While the subthreshold stimulus often induced somatic hyperpolarization, increasing the stimulation intensity consistently induced spiking (Fig. 3C, F). In line with previous modeling studies, our in silico results showed that extracellular stimulation elicits heterogeneous V_m_ changes in a compartment-specific manner. APs can be initiated in distal subcellular regions while the soma is hyperpolarized so that axonal/dendritic APs spread to the soma and overcome the initial local hyperpolarization, resulting in somatic firing^52^.

We next modeled the EF maps generated by each ISP slot of a rotating ISP stimulation pattern, consisting of nine different electrode configurations (Fig. 3D). The field vectors were extracted at five arbitrarily chosen anatomical locations (i.e., Mesencephalon, thalamus, visual cortex (at O1), parietal cortex (at P7), and at the Cz location). The nine vectors extracted at a location were used to construct the 9 ×100 µs long stimulation pattern applied in the NEURON simulations. We also constructed two tDCS equivalents of the ISP, with the magnitude of the vectorial and scalar mean of individual ISP field vectors and with their average direction (Fig. 3E). The model neurons were aligned to their least excitable orientation to the mean direction of the EF vectors, and their resting V_m_ was at −73 mV.

We determined the rheobase of the model neurons for all three stimulation types (i.e., ISP and its vectorial and scalar mean tDCSs) by measuring their V_m_ readouts at incrementally increased stimulus intensity (Fig. 3F, G).

During ISP stimulation, the spike threshold intensity was similar for neurons near the electrodes and those at targeted subcortical areas (229 ± 54.11 µC/ms vs 262 ± 130.87 µC/ms, *N* = 4 vs 6, for superficial and deep targets, respectively, Fig. 3H). In contrast, the application of the vectorial sum of the ISP-generated fields, resembling their simultaneous application as conventional TES, required 65 ± 5 % higher stimulation intensities to entrain deep structures compared to cortical areas proximal to the electrodes (387 ± 231.99 µC/ms vs 639 ± 221.34 µC/ms, *N* = 4 vs 6, for superficial vs deep targets, respectively, Fig. 3I). This finding supports the hypothesis that ISP can achieve more uniform stimulation across different brain depths.

Further, our model indicated that the neuronal response to ISP stimulation correlated better with the scalar sum of the individual ISP fields rather than their vectorial sum (Figs. 3H and 3I, R = 0.98, *p* < 0.001 for ISP vs scalar sum, and R = 0.71, *p* = 0.023 for ISP vs vectorial sum, *N* = 2 × 10 in both comparisons, Pearson’s linear correlation), providing computational support that neurons integrate ISP-induced fields in a non-vectorial manner.

To evaluate the spatial effect of ISP stimulation, we incorporated a universal rat head model^53^ into the ROAST toolbox^42,43^. We also simulated the volumetric EF distribution of our stimulation protocols in rats and channeled the EF readouts to the same neuronal models as above. These results were qualitatively comparable to the simulations performed on the human brain. They supported the non-vectorial summation of the ISP stimulation effects and better entrainment of deep structures than conventional TES (Supplementary Fig. 2).

### In vivo validation of non-vectorial field integration

**Fig. 4:**
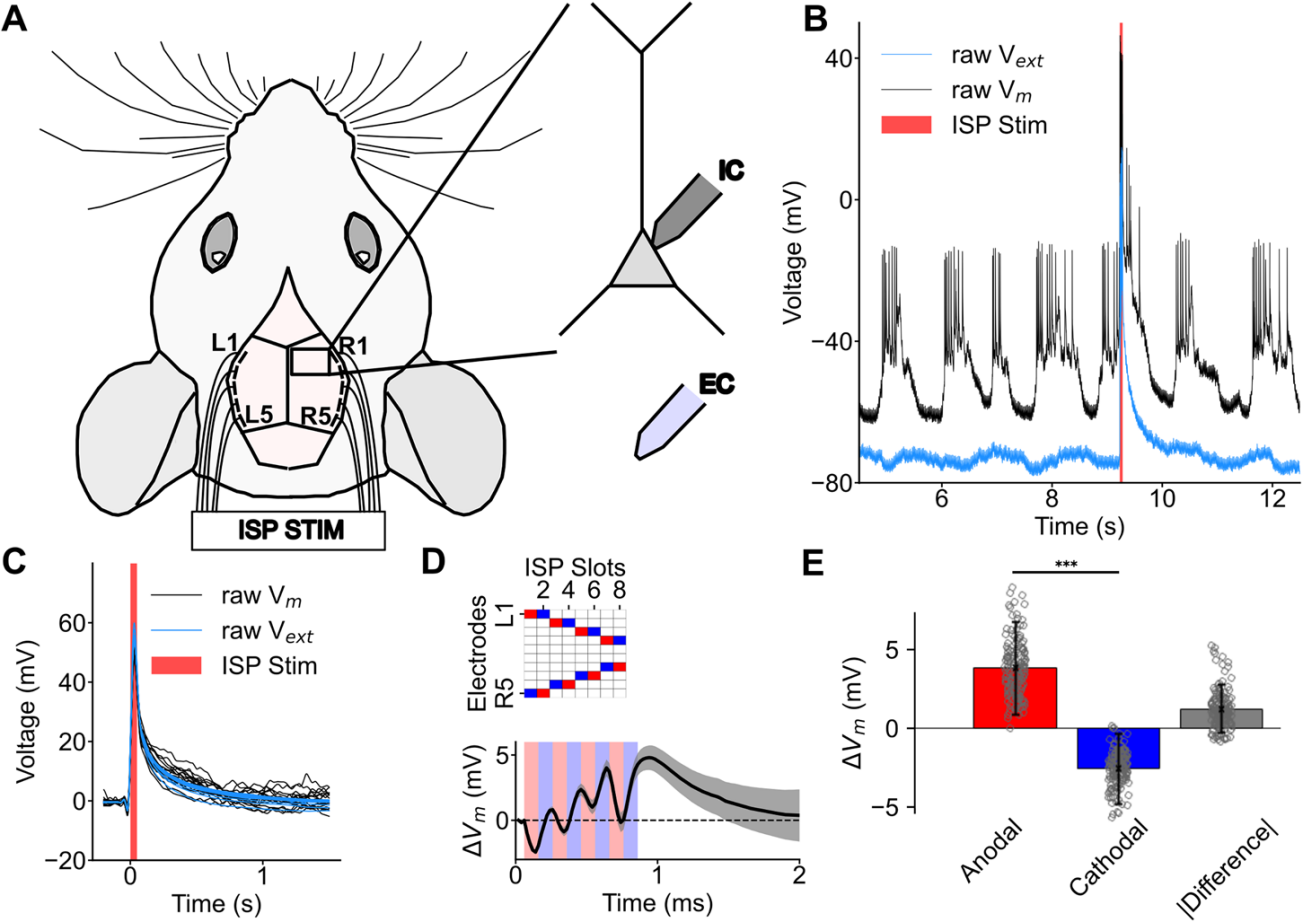
In vivo V_m_ recordings in rat neurons during symmetric ISP stimulation. **A** Experimental arrangement using five pairs (L1-L5 and R1-R5) of transcranial stimulation electrodes and an intracellular and subsequent extracellular recording. **B** Snapshot of the two-step measurement to eliminate the stimulation artifact. **C** Intra- (IC; black) and extracellularly (EC; blue) recorded voltage changes of the recorded cells triggered by ISP stimulation (red shaded area). **D** V_m_ changes induced by the individual ISP pulses. The table above denotes the stimulation sequence and electrode assignment. Note the perfectly symmetric and charge-balanced stimulation pattern. Blue: cathode, red: anode, white: disconnected. **E** The average V_m_ changes induced by the anodic and their mirrored cathodic pulses and their difference (right panel). Note the non-zero sum of symmetric pairs supporting a non-linear and non-vectorial summation. *P* < 0.001, paired t-test between the absolute values, *N* = 2×144 value pairs.

To validate our computational findings on the non-vectorial integration of the momentary effects of the subsequent EFs, we performed in vivo whole-cell patch-clamp recordings in layer 2/3 pyramidal neurons of the rat somatosensory cortex during ISP stimulation (Fig. 4A). We recorded intra- and extracellular potentials to calculate V_m_ changes in two cells, while concurrently applying ISP stimulation (Fig. 4B). The ISP stimulation sequence was designed to be symmetric and charge-balanced, i.e. alternating pulses and their evoked EFs had the same magnitude but inverse directions (Fig. 4D). For vectorial summation, the stimulus pair effects would cancel each other, and would not change the V_m_.

To eliminate the seconds-long ISP-induced electrical artifact from the electrophysiological recordings and evaluate its evoked net effect, the patch-clamp recordings were followed by extracellular recordings from the vicinity of the recorded neuron (Fig. 4A, B). In both configurations, ISP sequences were repeated 17–20 times and averaged (Fig. 4C). The mean extracellular voltage waveform was subtracted from the mean intracellular trace, eliminating the common mode stimulation artifact and resulting in the stimulation-induced net V_m_ change (Fig. 4D).

In both recorded cells, 0.8 ms of ISP stimulation (i.e. eight 100 μs-long ISP slots) at 100 μA triggered a net depolarizing effect, confirming that the symmetric stimulus pulse pairs induce non-symmetric changes in the V_m_ (Fig. 4D). Our analysis at a higher temporal resolution revealed that the anodic stimulus pulses elicited a stronger V_m_ change than its cathodic counterpart, resulting in a cumulative net depolarization (3.79 ± 2.94 mV for anodal and −2.58 ± 2.21 for cathodal stimulation, p < 0.001, paired t-test between the absolute values, N = 2 × 144 value pairs, Fig. 4D, E).

We applied the same modeling pipeline described previously to mechanistically analyze the neuronal responses to symmetric ISP stimulation. The instantaneous EFs induced by the individual electric pulses were simulated using ROAST (Supplementary Fig 3A) and subsequently incorporated into the NEURON model. The results revealed similar asymmetric V_m_ dynamics in response to symmetric stimulation, consistent with our in vivo measurements. A detailed analysis of the individual neuron segments showed that specific compartments were more sensitive to stimulation than the soma (Supplementary Fig. 3B). In many cases, stimulation near the cell’s rheobase intensity triggered APs in these distal segments rather than at the soma or axon hillock (Supplementary Fig. 3C).

These in vivo findings corroborate our computational results, supporting that integration of ISP pulse effects results from the cell membrane’s capacitive properties rather than extracellular integration of EFs. This integration is non-vectorial.

### Spatially focused ISP stimulation

**Fig. 5:**
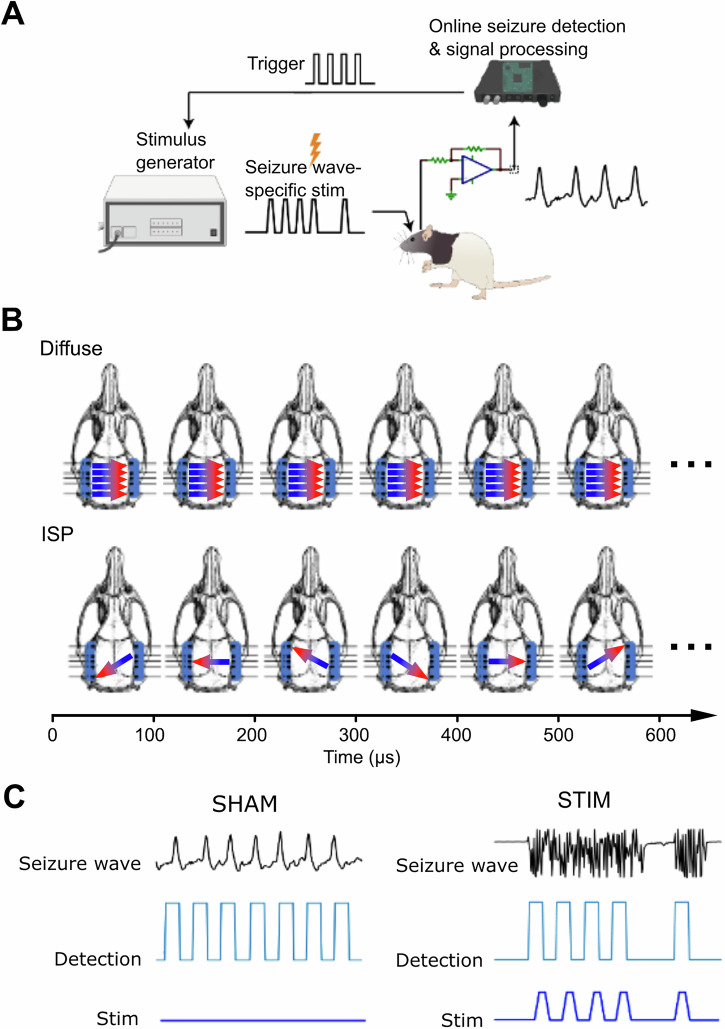
Closed-loop intervention and ISP stimulation sequence. **A** The concept of closed-loop seizure intervention through seizure rhythm-driven stimulation. Individual seizure waves were detected in real-time, and each detection triggered transcranial stimulation. **B** Stimulation protocol of diffuse TES compared to ISP. In the latter case, three electrode pairs were set to target the ISP pulses on either the left or the right hippocampus. Each electrode pair was pulsed for 100 μs, and the pulses cycled through the three pairs; this sequence was repeated to alternate between stimulating the right or left hemisphere, respectively. **C** A representative HPC seizure, its detection in the absence and presence of transcranial stimulation, left and right panels, respectively.

Previous findings demonstrated that precisely timed electrical stimulation (Fig. 5A) can efficiently interfere with neuronal oscillations and terminate thalamocortical seizures^1,45^. However, closed-loop diffuse TES was less effective in terminating tonic-clonic seizures in a bilateral TLE model. Our simulations support the hypothesis that ISP stimulation can generate EFs with comparable strength and direction in both hippocampi, which is difficult to achieve with conventional TES (Fig. 5B). In addition, the modeled field patterns across sequential ISP pulses generated electric vectors with varying orientations, which may enhance local efficacy in morphologically complex regions such as the hippocampus.

To test whether ISP can modulate seizure activity in vivo, we used hippocampus-kindled rats as a model of refractory TLE model^47^ and attempted to terminate stimulation-induced bilateral TLE seizures by inducing a closed-loop symmetric bilateral entrainment. Rats (*n* = 10) were kindled by daily electrical stimulation of the hippocampal commissure. The stimulation immediately provoked 10–25 Hz after-discharges bilaterally in the hippocampus (Fig. 5C). The kindled seizures were secondarily generalized (Racine’s score (RS) 5 motor seizures (10/10 rats). As positioning stimulating electrodes based on novel rat skull models^44^ can increase effective spatial targeting, we incorporated a universal rat head model^53^ in the ROAST toolbox^42,43,54^ to model the volumetric EF distribution of our stimulation (Supplementary Figs. 2 and 3).

We used two closed-loop transcranial stimulation paradigms to terminate seizures: conventional (diffuse) TES or ISP. TES protocols were applied between the left and right temporal screw electrodes, placed onto the frontal and temporal bone (Fig. 5B, upper panel). During ISP stimulation, we used six rotating dipoles to focus both hippocampi (Fig. 5B, lower panel). The stimulus pulse intensities were identical in both cases and were triggered by ictal LFP deflections in the hippocampus, resulting in continuous electrical stimulation throughout the ictal period (Fig. 5A, C). The signals were analyzed online to detect each LFP deflection in the HPC using a custom-made seizure detection algorithm built on an existing routine^45^. We applied traditional TES using trapezoidal waveforms following the detection of paroxysmal LFP events. The induced hippocampal seizures were detected online, and each HPC LFP deflection triggered a single-pulse stimulation delivered either as ISP or as conventional TES (+8 V trapezoid, 80 ms duration) (Fig. 5A, C).

### Efficacy of closed-loop ISP stimulation in terminating temporal lobe seizures

To test the efficacy of ISP in controlling pathological brain activity, we employed a hippocampal kindling model of TLE using male Long-Evans rats kindled by daily electrical hippocampal commissure stimulation until they consistently exhibited stage 5 RS seizures. We compared the effects of closed-loop ISP stimulation to conventional TES and SHAM stimulation on seizure duration and severity.

**Fig. 6:**
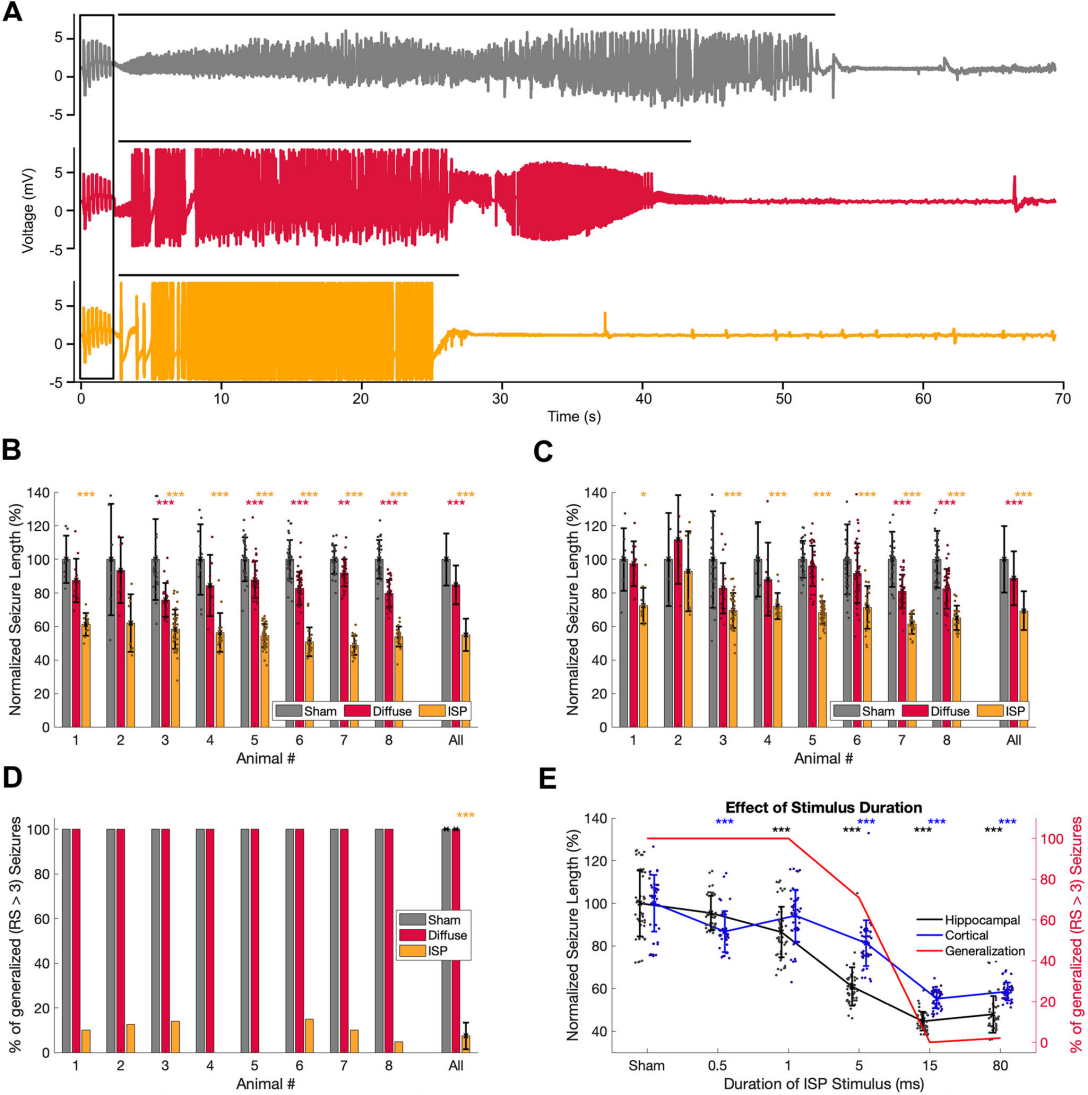
Anti-epileptic effects of ISP stimulation. **A** Representative LFP traces of HPC seizures induced by kindling stimulation with or without closed-loop transcranial stimulation. The black bracket denotes the kindling epochs, while horizontal black lines represent detected seizures. HPC (**B**) and CTX (**C**) seizure durations during SHAM (grey), DIFFUSE (red), and ISP (orange) stimulation, normalized to the mean duration of the SHAM-stimulated seizures within each animal, and the percentage of generalized > 3 RS seizures (**D**). Data are presented both as per animal and pooled averages. Scatter plots denote the data points of the individual seizures (*n* = 166, 174 and 192 seizures per condition [SHAM, DIFFUSE, ISP], recorded across 8 animals; statistical comparisons were performed on a per-seizure basis using two-sample t-tests against the SHAM conditions, with Bonferroni correction). **E** HPC and CTX seizure durations, in black and blue, respectively, and the percent of generalized > 3 RS seizures as a function of ISP stimulus duration. Within animal normalization was performed to the mean seizure duration of the SHAM stimulated seizures (*n* = 48 seizures for each stimulus duration from two animals; statistical comparisons were performed on a per-seizure basis). The number of recorded seizures for each condition and animal is reported in Supplementary Table 1.

Closed-loop ISP stimulation significantly reduced normalized hippocampal seizure duration compared to SHAM (55.08 ± 9.67% vs 100 ± 15.52%, p < 0.001) and conventional TES (55 ± 0.8% vs 84.9 ± 11.6%, *p* < 0.001) (Fig. 6B, paired t test with Bonferroni correction). Similar results were observed for cortical seizure duration (Fig. 6C).

ISP stimulation substantially reduced the severity (RS) of seizures (prevalence of RS > 3 seizures: ISP: 7.47 ± 5.97 %, diffuse TES: 100 %, SHAM: 100 %; ***p < 0.001 paired t-tests with Bonferroni correction) (Fig. 6D). This marked reduction in seizure severity demonstrates the superior efficacy of ISP in reducing seizure intensity compared to traditional TES.

In two additional animals, we tested the effect of varying pulse durations on the efficacy of ISP stimulation. Decreasing stimulation duration from 80 to 15 ms did not affect efficacy, while below 15 ms, effectiveness decreased (Fig. 6E). This optimization allows minimizing stimulation duration to achieve the same effect but reducing unwanted increased stimulation of individual sites, thus decreasing side effects and improving tolerability.

Together, these results demonstrate that closed-loop ISP stimulation can rapidly and effectively decrease the duration and severity of temporal lobe seizures more effectively than conventional TES, further underlying its potential as a tool for deep brain stimulation.

## Discussion

Our data demonstrate the superior efficacy of Intersectional Short-Pulse (ISP) stimulation in terminating epileptic seizures and begin to elucidate its mechanistic differences from conventional transcranial electrical stimulation (TES) in entraining neuronal activity. Our simulations, cadaver measurements, and in vivo recordings support improved spatial focusing and efficacy of ISP versus conventional TES, particularly for terminating temporal lobe seizures. We propose that a key mechanism underlying ISP’s effectiveness is the non-vectorial integration of induced electric fields (EF) by neurons, which is predicted to allow deeper and more uniform stimulation while minimizing peripheral side effects. Importantly, our seizure experiments were not designed to distinguish among candidate network-level mechanisms (e.g., focal desynchronization, nonspecific perturbation, or transient depolarization block), so these remain speculative at this stage.

The limitations of conventional TES, including poor spatial focality, shallow depth penetration, and peripheral side effects, have driven the development of various innovations aimed at enhancing its efficacy and tolerability. Among the most notable advancements are Temporal Interference (TI) stimulation and high-definition transcranial direct current stimulation (HD-tDCS), each of which employs unique principles to address these challenges.

Similar to ISP, TI stimulation, as proposed by Grossman and colleagues^36^, also utilizes the non-linearity of neuronal cell membranes^38^. However, TI is stimulated by the interference patterns of two concurrently applied high-frequency signals through separate electrode pairs. The interaction of these currents creates a low-frequency EF at a targeted region, enabling low-frequency entrainment of deeper brain structures while exposing the superficial layers only to the high-frequency pattern. Clinical applications of TI have shown promise in treating conditions such as depression^37,55^ and have been proven to modulate brain activity in various models of health and disease^56–58^. However, TI relies heavily on precise electrode placement and stable interference conditions, which can be challenging in practical settings. Additionally, the high-frequency components can induce peripheral nerve stimulation, limiting tolerable intensities and constraining effective dose delivery to deeper targets.

High-definition tDCS, on the other hand, improves the spatial resolution of traditional tDCS by utilizing multi-electrode array configurations. This approach confines the EF to smaller cortical areas, enhancing focality and reducing off-target effects^31^. HD-tDCS has demonstrated its utility in modulating cortical excitability for cognitive enhancement and therapeutic applications, including stroke rehabilitation and depression^59–66^. Despite these advantages, HD-tDCS struggles with efficiently stimulating deeper brain structures.

In contrast, ISP stimulation uses a paradigm that combines temporal and spatial principles. By delivering rapid sequences of brief electric pulses through dynamically switching electrode pairs, ISP exploits the neuronal membrane’s capacity for temporal integration. This scalar integration mechanism enables the ISP to achieve increased penetration into deep brain structures. Additionally, ISP does not introduce any constraint on the stimulation waveform envelope, allowing for precise temporal targeting of complex neuronal oscillations. The temporal and spatial distribution of charge injection reduces peripheral discomfort, facilitating the delivery of higher stimulation intensities and improving overall efficacy, making it suitable for phase-matched closed-loop applications such as seizure termination.

To further advance ISP and make informed decisions regarding optimal stimulation patterns and electrode configurations, a validated modeling pipeline is necessary. Additionally, a mechanistic understanding of how ISP interacts with neurons is critical for refining its application and maximizing its therapeutic potential.

As a validation step, we compared the predictions of the ROAST (Realistic volumetric approach to simulate transcranial electric stimulation) finite element modeling algorithm against actual measurements in human cadavers during ISP stimulation. This validation was essential to ensure computational model reliability in our ISP high-frequency regime.

The strong correlation between the modeled and measured voltage gradients provides robust support for the ROAST in simulating ISP-induced EFs. This high degree of concordance across various stimulation patterns and brain regions, with accurate predictions for larger induced EF gradients.

While the predicted EF distributions were qualitatively similar to the measured distributions, quantitatively, the modelling was consistently slightly overestimating the EF strengths. This may stem from the finite element model of ROAST, lacking the silicone insulation around the electrode contacts and the slightly altered conductivity of the post-mortem tissues^22^.

The model predictions were less precise for stimulations between closely spaced electrodes, likely due to the local tissue shunting effects and the model’s sensitivity to exact tissue segmentation. This observation highlights the importance of electrode placement when designing ISP protocols and interpreting simulation results.

The consistent correlation coefficients observed for ISP pairs with swapped electrode polarities further underscore the reliability of the ROAST framework for modeling transient EFs. Swapping polarities also reduces undesirable side effects, such as scalp burning^22^. These findings suggest that the model accurately captures the fundamental physics of current flow in the brain, regardless of the specific electrode configuration.

Contrary to the expectation that increasing extracellular EF intensity would linearly enhance neuronal depolarization, in our modeling work, we observed an initial somatic hyperpolarization under weak EFs that transitioned to AP initiation as the EF intensity increased. This poses the issue of how mild hyperpolarization can lead to spiking with elevated stimulation. Excitable neurons exhibit a non-linear response to EFs, where the effect of the EF on neuronal activity depends on the intensity of the field, neuronal morphology, and ion channel composition of the neuronal membrane. At subthreshold EF intensities, passive polarization can cause somatic hyperpolarization and distal depolarization (e.g., distal axonal segments or tuft dendrites). This occurs because neuronal compartments respond differentially to EFs due to variations in geometry and ion channel distributions. According to the model, as the EF intensity increases, the depolarization at excitable regions, such as the Ranvier nodes of distal axonal compartments, can reach the threshold for AP initiation, leading to antidromic spiking even if the soma remains hyperpolarized.

Importantly, a recent human cortical study demonstrated that single-pulse electrical stimulation broadly suppressed firing rates but elicited facilitation in specific neuronal populations depending on distance from the electrode and cell-type characteristics. This finding provides strong empirical support for the non-linear and spatially heterogeneous effects of extracellular fields predicted by our model^67^.

This non-linear readout underscores the need to consider spatially heterogeneous effects of EFs on neurons. The timing and spatial distribution of EFs significantly influence neuronal excitability^68^, highlighting that weak fields can modulate neuronal activity when they interact with intrinsic neuronal properties. This emphasizes the complex interplay between extracellular fields and neuronal structures, with implications for understanding neural processing and for developing neuromodulation therapies.

Our computational modeling and in vivo patch-clamp recordings provide converging evidence that neurons integrate ISP-induced EFs in a scalar rather than vectorial manner. This finding departs from the traditional understanding of how neurons respond to externally applied EFs. The scalar integration may help ISP to overcome the “mirror effect” that limits conventional TES, where simultaneous activation of surface electrodes induces opposing effects under cathode and anode placements^25^.

Several key observations from our study support the scalar integration hypothesis. Our computational models revealed that the neuronal response to ISP stimulation correlated strongly with the scalar sum of individual ISP fields rather than their vectorial sum. This finding was corroborated by in vivo patch-clamp recordings, which showed that the scalar sum of pulse intensities more precisely explains the integrated effect of ISP stimulation. Further, the intensity threshold for evoking APs was similar for superficial and deep brain structures during ISP stimulation, in contrast to the depth-dependent effects seen with conventional TES. Collectively, these findings suggest that ISP pulse integration effects occur primarily due to the capacitive properties of the cell membrane rather than the instantaneous summation of extracellular EFs. This mechanism allows the ISP to achieve more uniform stimulation at different brain depths, an advantage for targeting deep brain structures such as the hippocampus.

While our study primarily focused on single-neuron responses, the robust seizure-suppressing effect of ISP implies that it perturbs neural networks and brain oscillations as well. Based on our modeling, ISP can generate multidirectional EFs in target structures that we hypothesize to disrupt synchronization across neuronal populations. By influencing the timing and probability of neuronal firing in a spatially focused manner, ISP could reset the phase of ongoing oscillations and limit the spread of epileptiform activity. This hypothesized network-level impact may be a crucial link between the observed cellular effects and the macroscopic seizure control and will require direct electrophysiological validation.

The enhanced efficacy of closed-loop ISP stimulation in terminating seizures may result from precise temporal alignment with ongoing seizure rhythms. This timing could enable destructive interference with the pathological oscillations^1,69^. By delivering focused, multidirectional stimulation pulses at specific phases of the seizure rhythm, ISP may disrupt the spatial and temporal coherence necessary for seizure maintenance and propagation. We hypothesize that the scalar integration of ISP-induced fields by neurons results in a more robust and widespread disruption of hypersynchronous activity compared to conventional TES. The rapid cycling of ISP stimulation sites may limit adaptive changes in neural networks that could reduce standard TES stimulation efficacy over time.

Notably, the therapeutic seizure termination observed in our experiments required higher stimulation intensities compared to those employed in intracellular measurements. This indicates that, beyond subthreshold modulation of neuronal excitability, direct activation of neurons by individual ISP pulses may also contribute to seizure control. Such pulse-evoked firing could disrupt pathological synchrony or induce transient depolarization block or other nonspecific perturbations of local circuits, thereby enhancing termination. These findings highlight the importance of investigating the intensity-dependent effects of ISP in greater detail. While our data qualitatively demonstrate the mechanistic link between ISP stimulation and seizure suppression, establishing a quantitative correlation between local electric field magnitude and therapeutic efficacy will require future studies combining high-density field mapping with behavioral readouts.

Our simulations and intracranial measurements show that ISP trains can steer the loci of effective neuromodulation toward bilateral deep targets - such as the hippocampi - more effectively than conventional TES in our models and experimental configuration. While the raw EF amplitude still decays between cortex and hippocampus, consistent with CSF and tissue shunting^22,70–72^, the enhanced deep entrainment is supported by two factors. On one hand, ISP’s rapid, charge-balanced pulses raise the tolerable scalp current relative to lower frequency or monotonic waveforms^73,74^. On the other hand, neurons scalar-integrate the cumulative charge delivered over the 100-µs pulse sequence, a principle established for TI and other kHz paradigms^36^. Importantly, membrane-level temporal integration is not exclusive to deep structures: superficial cortical neurons can also integrate repeated ISP exposures when the repetition rate is fast relative to their membrane time constant. However, because ISP distributes its brief pulses across many rapidly alternating electrode configurations, no single superficial region receives a temporally concentrated drive. Consequently, despite weaker instantaneous fields, deep neurons are modulated by the integral effect of the rapid EFs induced by the ISP train, while superficial electrodes share the charge load, reducing peripheral sensations and permitting higher intensities.  This depth-competent, multi-axial steering can support network-level interventions, for example, bilateral hippocampal engagement in TLE. However, the inter-individual variability of cranial geometry, anatomy, and nociceptive sensitivity, as well as the inhomogeneities of tissue conductance, may require personalized modelling to achieve consistent effects^72,75^.

The marked reduction in seizure duration and severity achieved by closed-loop ISP stimulation in our rat TLE model underscores ISP’s therapeutic potential and compares it favorably with other non-invasive approaches^58,76^. The persisting therapeutic effect with shortened pulse duration to 15 µs allows further optimization of ISP protocols to minimize cutaneous side-effects without sacrificing efficacy, underscoring their translational potential.

While our findings in the rat TLE model are promising, translating these results to humans is challenging. The human brain is larger with complex folding, making focused stimulation in deep structures more difficult. However, the scalar integration in our study should also apply to human neurons, suggesting that ISP could maintain its advantages over conventional TES in human applications. Computational models of ISP in human head models, followed by carefully designed clinical trials, are the next crucial steps. Initial human studies might focus on individuals with refractory epilepsy who are candidates for invasive treatments, allowing for direct comparison of ISP with other interventions. Our closed-loop ISP paradigm aligns well with emerging trends in personalized medicine, potentially allowing for tailored stimulation parameters based on individual seizure patterns.

Moving to human trials, we need to optimize ISP parameters for the human brain, assess its safety profile over extended use, and explore its potential in treating other neurological and psychiatric disorders involving aberrant neural synchronization. The promising results of ISP stimulation in controlling epileptic seizures open up several avenues for clinical application and further research. ISP could be a non-invasive alternative or adjunct to treat drug-resistant epilepsy, particularly for patients who are not candidates for surgical interventions or who have not benefited from existing surgical and neuromodulatory approaches. Beyond epilepsy, non-invasive ISP for focused, deep brain stimulation could help treat neuropsychiatric disorders involving limbic and extrapyramidal structures, including major depressive disorder, anxiety disorders, including obsessive-compulsive disorder, and Parkinson’s disease.

Future studies should explore the potential for patient-specific ISP protocols, optimizing electrode placement and stimulation parameters based on individual brain anatomy and pathology. Integrating ISP with individual structural and functional neuroimaging could further enhance the targeting for spatial and temporal precision. Future developments may also include adaptive closed-loop algorithms that integrate scalp- or subgaleal-level detection to dynamically adjust ISP parameters in real time, potentially improving efficacy across different seizure states and patient-specific conditions. While our study focused on acute effects, future research should investigate the long-term impacts of ISP stimulation on brain plasticity and the potential beneficial as well as adverse effects.

Despite the promising results, several limitations of our study require further research. While the rat kindling model is well-established, studies in larger animals and eventually humans are necessary to confirm or refute the translational potential of ISP. Although we propose scalar integration as the primary mechanism, further investigation into the cellular and network-level effects of ISP is warranted. While we explored pulse duration, other parameters, such as frequency, intensity, and electrode configuration, could be further optimized. Finally, a direct comparison of ISP with other emerging TES approaches, such as TI stimulation, is needed to fully assess its relative advantages and potential clinical utility.

In conclusion, ISP stimulation is a significant advancement in non-invasive brain stimulation. By exploiting the scalar integration of induced EFs by neurons, ISP improves spatial focusing and efficacy in modulating neural activity, particularly in deep brain structures. Our ability to rapidly terminate temporal lobe seizures in a rat model highlights its compelling therapeutic potential. With additional refinements and validation in humans, ISP stimulation may open new avenues to treat diverse neurological and psychiatric disorders, offering hope for patients who have not responded to conventional therapies.

## Supplementary information

Supplementary Information

Description of Additional Supplementary Files

Supplementary Data 1

## Data Availability

The data generated in this study are available from the corresponding author upon reasonable request. The numerical results underlying the graphs and charts presented in the main figures are available in Supplementary Data 1.

## Code availability

All custom codes are freely available from the corresponding author on reasonable request.
